# *catena*-Poly[[{2-[(2-hy­droxy­eth­yl)imino­meth­yl]-6-meth­oxy­phenolato}copper(II)]-μ-thio­cyanato]

**DOI:** 10.1107/S1600536810034021

**Published:** 2010-08-28

**Authors:** Ling-Wei Xue, Gan-Qing Zhao, Yong-Jun Han, Yun-Xiao Feng

**Affiliations:** a College of Chemistry and Chemical Engineering, Pingdingshan University, Pingdingshan, Henan 467000, People’s Republic of China

## Abstract

In the title thio­cyanate-bridged polynuclear copper(II) complex, [Cu(C_10_H_12_NO_3_)(NCS)]_*n*_, the Cu atom is five-coordinated in a square-pyramidal geometry, with one phenolato O, one imino N and one hy­droxy O atom of a Schiff base ligand and one thio­cyanato N atom defining the basal plane, and with one thio­cyanato S atom occupying the apical position. In the crystal structure, pairs of adjacent complex mol­ecules are linked through inter­molecular O—H⋯O hydrogen bonds into dimers. The dimers are further linked *via* Cu⋯S inter­actions, forming two-dimensional layers parallel to the *bc* plane.

## Related literature

For the biological properties of Schiff bases, see: Bhandari *et al.* (2008 ▶); Sinha *et al.* (2008 ▶); Sondhi *et al.* (2006 ▶); Singh *et al.* (2006 ▶). For metal complexes with Schiff bases, see: Assey *et al.* (2010 ▶); Thiam *et al.* (2010 ▶); Montazerozohori *et al.* (2009 ▶); Eltayeb *et al.* (2009 ▶).
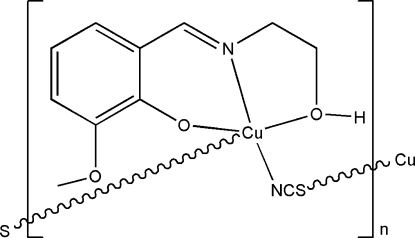


## Experimental

### 

#### Crystal data

[Cu(C_10_H_12_NO_3_)(NCS)]
*M* *_r_* = 315.83
Monoclinic, 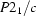

*a* = 10.123 (2) Å
*b* = 11.812 (2) Å
*c* = 10.264 (2) Å
β = 94.122 (2)°
*V* = 1224.1 (4) Å^3^
*Z* = 4
Mo *K*α radiation
μ = 1.96 mm^−1^
*T* = 298 K
0.23 × 0.20 × 0.20 mm

#### Data collection

Bruker SMART CCD area-detector diffractometer
Absorption correction: multi-scan (*SADABS*; Sheldrick, 1996 ▶) *T*_min_ = 0.662, *T*_max_ = 0.696
6414 measured reflections
2602 independent reflections
1875 reflections with *I* > 2σ(*I*)
*R*_int_ = 0.043

#### Refinement

*R*[*F* ^2^ > 2σ(*F* ^2^)] = 0.046
*wR*(*F* ^2^) = 0.097
*S* = 1.04
2602 reflections
167 parameters
1 restraint
H atoms treated by a mixture of independent and constrained refinement
Δρ_max_ = 0.36 e Å^−3^
Δρ_min_ = −0.39 e Å^−3^

### 

Data collection: *SMART* (Bruker, 1998 ▶); cell refinement: *SAINT* (Bruker, 1998 ▶); data reduction: *SAINT*; program(s) used to solve structure: *SHELXS97* (Sheldrick, 2008 ▶); program(s) used to refine structure: *SHELXL97* (Sheldrick, 2008 ▶); molecular graphics: *SHELXTL* (Sheldrick, 2008 ▶); software used to prepare material for publication: *SHELXTL*.

## Supplementary Material

Crystal structure: contains datablocks global, I. DOI: 10.1107/S1600536810034021/om2356sup1.cif

Structure factors: contains datablocks I. DOI: 10.1107/S1600536810034021/om2356Isup2.hkl

Additional supplementary materials:  crystallographic information; 3D view; checkCIF report

## Figures and Tables

**Table 1:** Selected bond lengths (Å)

Cu1—O1	1.902 (2)
Cu1—N1	1.910 (3)
Cu1—N2	1.933 (3)
Cu1—O2	2.035 (3)
Cu1—S1^i^	2.983 (3)

Symmetry code: (i) 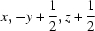.

**Table 2:** Hydrogen-bond geometry (Å, °)

*D*—H⋯*A*	*D*—H	H⋯*A*	*D*⋯*A*	*D*—H⋯*A*
O2—H2⋯O1^ii^	0.85 (1)	1.96 (2)	2.770 (4)	160 (5)
O2—H2⋯O3^ii^	0.85 (1)	2.41 (4)	3.020 (4)	129 (4)

Symmetry code: (ii) 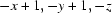.

**Fig. 1.**
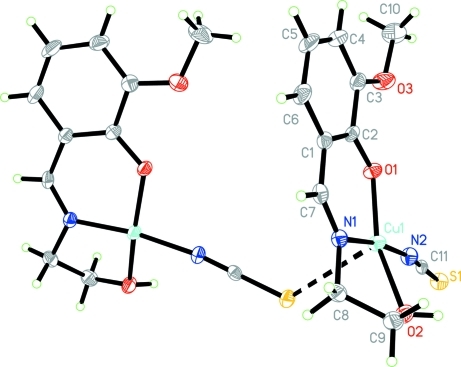
The structure of the title complex with 30% probability displacement ellipsoids.

**Fig. 2.**
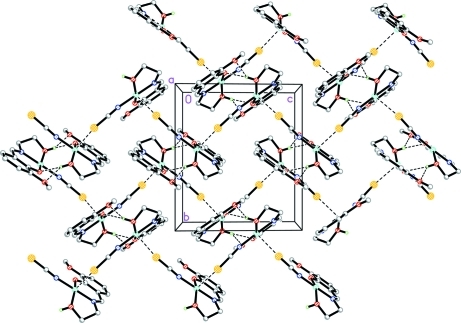
The molecular packing of the title complex. Intermolecular hydrogen bonds are shown as dashed lines. H atoms unrelated to the hydrogen bonding have been omitted for clarity.

## supplementary crystallographic information

### Comment

Schiff bases are a kind of versatile compounds, which possess excellent biological properties (Bhandari *et al.*, 2008; Sinha *et al.*, 2008; Sondhi *et al.*, 2006; Singh *et al.*, 2006). The metal complexes derived from Schiff bases have been extensively studied (Assey *et al.*, 2010; Thiam *et al.*, 2010; Montazerozohori *et al.*, 2009; Eltayeb *et al.*, 2009). In this paper, a new thiocyanato-bridged polynuclear copper(II) complex with the Schiff base 2-[(2-hydroxyethylimino)methyl]-6-methoxyphenol is reported.

The complex is a thiocyanato-bridged polynuclear copper(II) complex, as shown in Fig. 1. The Cu atom in the complex is five-coordinate in a square pyramidal geometry, with one phenolate O, one imine N, and one hydroxy O atoms of a Schiff base ligand, and with one thiocyanate N atom, occupying the basal plane, and with one thiocyanate S atom occupying the apical position. The Cu···S' (S' at *x*, 1/2 - *y*, 1/2 + *z*) distance is 2.983 (3) Å. The Cu atom displaced 0.141 (2) Å from the plane defined by the four basal donor atoms. The slight distortion of the square pyramidal coordination can be observed from the coordinate bond lengths and angles (Table 1).

In the crystal structure, the adjacent complex molecules are linked through intermolecular O—H···O hydrogen bonds (Table 2), to form a dimer. The dimers are further linked *via* Cu···S interactions, forming a two-dimensional layers parallel to the *bc* plane (Fig. 2).

### Experimental

3-Methoxysalicylaldehyde (152.1 mg, 1.0 mmol), 2-aminoethanol (61.1 mg, 1.0 mmol), ammonium thiocyanate (76.0 mg, 1.0 mmol), and copper acetate monohydrate (199.2 mg, 1.0 mmol) were dissolved in methanol (80 ml). The mixture was stirred for two hours at room temperature. The resulting solution was left in air for a few days, yielding blue block-like crystals.

### Refinement

H2 was located in a difference Fourier map and refined isotropically, with O—H distance restrained to 0.85 (1) Å, and with *U*_iso_(H) fixed at 0.08 Å^2^. Other H atoms were placed in idealized positions and constrained to ride on their parent atoms with C—H distances of 0.93–0.97 Å, and with *U*_iso_(H) set at 1.2*U*_eq_(C) and 1.5*U*_eq_(C10).

### Crystal data

**Table:**

[Cu(C_10_H_12_NO_3_)(NCS)]	*F*(000) = 644
*M**_r_* = 315.83	*D*_x_ = 1.714 Mg m^−^^3^
Monoclinic, *P*2_1_/*c*	Mo *K*α radiation, λ = 0.71073 Å
*a* = 10.123 (2) Å	Cell parameters from 1160 reflections
*b* = 11.812 (2) Å	θ = 2.5–24.5°
*c* = 10.264 (2) Å	µ = 1.96 mm^−^^1^
β = 94.122 (2)°	*T* = 298 K
*V* = 1224.1 (4) Å^3^	Block, blue
*Z* = 4	0.23 × 0.20 × 0.20 mm

### Data collection

**Table:**

Bruker SMART CCD area-detector diffractometer	2602 independent reflections
Radiation source: fine-focus sealed tube	1875 reflections with *I* > 2σ(*I*)
graphite	*R*_int_ = 0.043
ω scans	θ_max_ = 27.0°, θ_min_ = 2.6°
Absorption correction: multi-scan (*SADABS*; Sheldrick, 1996)	*h* = −12→12
*T*_min_ = 0.662, *T*_max_ = 0.696	*k* = −15→9
6414 measured reflections	*l* = −10→13

### Refinement

**Table:**

Refinement on *F*^2^	Primary atom site location: structure-invariant direct methods
Least-squares matrix: full	Secondary atom site location: difference Fourier map
*R*[*F*^2^ > 2σ(*F*^2^)] = 0.046	Hydrogen site location: inferred from neighbouring sites
*wR*(*F*^2^) = 0.097	H atoms treated by a mixture of independent and constrained refinement
*S* = 1.04	*w* = 1/[σ^2^(*F*_o_^2^) + (0.0374*P*)^2^ + 0.2873*P*] where *P* = (*F*_o_^2^ + 2*F*_c_^2^)/3
2602 reflections	(Δ/σ)_max_ < 0.001
167 parameters	Δρ_max_ = 0.36 e Å^−^^3^
1 restraint	Δρ_min_ = −0.39 e Å^−^^3^

### Special details

**Table:**

Geometry. All e.s.d.'s (except the e.s.d. in the dihedral angle between two l.s. planes) are estimated using the full covariance matrix. The cell e.s.d.'s are taken into account individually in the estimation of e.s.d.'s in distances, angles and torsion angles; correlations between e.s.d.'s in cell parameters are only used when they are defined by crystal symmetry. An approximate (isotropic) treatment of cell e.s.d.'s is used for estimating e.s.d.'s involving l.s. planes.
Refinement. Refinement of *F*^2^ against ALL reflections. The weighted *R*-factor *wR* and goodness of fit *S* are based on *F*^2^, conventional *R*-factors *R* are based on *F*, with *F* set to zero for negative *F*^2^. The threshold expression of *F*^2^ > σ(*F*^2^) is used only for calculating *R*-factors(gt) *etc*. and is not relevant to the choice of reflections for refinement. *R*-factors based on *F*^2^ are statistically about twice as large as those based on *F*, and *R*- factors based on ALL data will be even larger.

### Fractional atomic coordinates and isotropic or equivalent isotropic displacement parameters (Å^2^)

**Table:**

	*x*	*y*	*z*	*U*_iso_*/*U*_eq_	
Cu1	0.53293 (4)	0.44688 (4)	0.14711 (4)	0.03113 (16)	
N1	0.5018 (3)	0.5378 (2)	0.2957 (3)	0.0287 (7)	
N2	0.5887 (3)	0.3555 (3)	0.0048 (3)	0.0347 (8)	
O1	0.3542 (2)	0.3978 (2)	0.1187 (2)	0.0313 (6)	
O2	0.6958 (2)	0.5474 (2)	0.1426 (3)	0.0369 (7)	
O3	0.1241 (2)	0.3292 (2)	0.0317 (3)	0.0502 (8)	
S1	0.69266 (9)	0.20646 (9)	−0.17233 (10)	0.0367 (3)	
C1	0.2666 (3)	0.5001 (3)	0.2971 (4)	0.0299 (9)	
C2	0.2552 (3)	0.4302 (3)	0.1865 (4)	0.0282 (8)	
C3	0.1256 (4)	0.3941 (3)	0.1410 (4)	0.0356 (9)	
C4	0.0163 (4)	0.4253 (4)	0.2056 (4)	0.0467 (11)	
H4	−0.0676	0.4013	0.1746	0.056*	
C5	0.0312 (4)	0.4923 (4)	0.3164 (5)	0.0512 (12)	
H5	−0.0428	0.5120	0.3602	0.061*	
C6	0.1528 (4)	0.5294 (4)	0.3616 (4)	0.0423 (11)	
H6	0.1613	0.5747	0.4358	0.051*	
C7	0.3894 (4)	0.5518 (3)	0.3442 (3)	0.0308 (9)	
H7	0.3874	0.5995	0.4161	0.037*	
C8	0.6186 (4)	0.6025 (3)	0.3450 (4)	0.0353 (10)	
H8A	0.6777	0.5550	0.3997	0.042*	
H8B	0.5922	0.6665	0.3964	0.042*	
C9	0.6868 (4)	0.6430 (3)	0.2283 (4)	0.0394 (10)	
H9A	0.6362	0.7034	0.1844	0.047*	
H9B	0.7744	0.6714	0.2551	0.047*	
C10	−0.0026 (4)	0.2961 (4)	−0.0268 (5)	0.0643 (15)	
H10A	−0.0477	0.2509	0.0338	0.096*	
H10B	0.0091	0.2528	−0.1043	0.096*	
H10C	−0.0542	0.3624	−0.0492	0.096*	
C11	0.6318 (3)	0.2926 (3)	−0.0683 (4)	0.0265 (8)	
H2	0.701 (5)	0.568 (4)	0.0643 (18)	0.080*	

### Atomic displacement parameters (Å^2^)

**Table:**

	*U*^11^	*U*^22^	*U*^33^	*U*^12^	*U*^13^	*U*^23^
Cu1	0.0286 (2)	0.0349 (3)	0.0305 (3)	−0.0015 (2)	0.00595 (18)	−0.0093 (2)
N1	0.0324 (16)	0.0280 (19)	0.0260 (17)	0.0028 (13)	0.0036 (13)	−0.0012 (14)
N2	0.0380 (17)	0.038 (2)	0.0291 (19)	0.0019 (15)	0.0067 (14)	−0.0066 (16)
O1	0.0285 (13)	0.0328 (15)	0.0335 (16)	−0.0030 (11)	0.0085 (11)	−0.0096 (12)
O2	0.0373 (14)	0.0396 (18)	0.0348 (16)	−0.0047 (12)	0.0100 (13)	−0.0066 (15)
O3	0.0379 (15)	0.060 (2)	0.052 (2)	−0.0168 (14)	0.0020 (13)	−0.0150 (17)
S1	0.0390 (5)	0.0337 (6)	0.0376 (6)	0.0032 (4)	0.0050 (4)	−0.0110 (5)
C1	0.0325 (19)	0.030 (2)	0.028 (2)	0.0055 (16)	0.0064 (16)	0.0026 (18)
C2	0.0301 (18)	0.024 (2)	0.032 (2)	−0.0017 (16)	0.0092 (16)	0.0061 (17)
C3	0.038 (2)	0.033 (2)	0.035 (2)	−0.0047 (18)	0.0054 (18)	0.005 (2)
C4	0.031 (2)	0.060 (3)	0.050 (3)	−0.004 (2)	0.0109 (19)	0.008 (2)
C5	0.039 (2)	0.067 (3)	0.051 (3)	0.008 (2)	0.026 (2)	0.008 (3)
C6	0.045 (2)	0.048 (3)	0.036 (2)	0.011 (2)	0.0183 (19)	0.002 (2)
C7	0.043 (2)	0.028 (2)	0.022 (2)	0.0052 (17)	0.0034 (16)	−0.0042 (18)
C8	0.037 (2)	0.034 (2)	0.035 (2)	−0.0026 (17)	−0.0045 (17)	−0.0041 (19)
C9	0.038 (2)	0.037 (3)	0.042 (3)	−0.0071 (18)	−0.0020 (18)	0.000 (2)
C10	0.050 (3)	0.067 (3)	0.075 (4)	−0.029 (2)	−0.008 (2)	−0.005 (3)
C11	0.0287 (18)	0.028 (2)	0.022 (2)	−0.0040 (16)	−0.0017 (15)	0.0055 (18)

### Geometric parameters (Å, °)

**Table:**

Cu1—O1	1.902 (2)	C2—C3	1.426 (5)
Cu1—N1	1.910 (3)	C3—C4	1.380 (5)
Cu1—N2	1.933 (3)	C4—C5	1.385 (6)
Cu1—O2	2.035 (3)	C4—H4	0.9300
Cu1—S1^i^	2.983 (3)	C5—C6	1.357 (6)
N1—C7	1.285 (4)	C5—H5	0.9300
N1—C8	1.467 (4)	C6—H6	0.9300
N2—C11	1.164 (4)	C7—H7	0.9300
O1—C2	1.317 (4)	C8—C9	1.502 (5)
O2—C9	1.439 (5)	C8—H8A	0.9700
O2—H2	0.846 (10)	C8—H8B	0.9700
O3—C3	1.357 (5)	C9—H9A	0.9700
O3—C10	1.431 (4)	C9—H9B	0.9700
S1—C11	1.627 (4)	C10—H10A	0.9600
C1—C2	1.401 (5)	C10—H10B	0.9600
C1—C6	1.413 (5)	C10—H10C	0.9600
C1—C7	1.438 (5)		
			
O1—Cu1—N1	94.82 (11)	C5—C4—H4	119.9
O1—Cu1—N2	92.31 (11)	C6—C5—C4	120.5 (4)
N1—Cu1—N2	172.47 (12)	C6—C5—H5	119.7
O1—Cu1—O2	159.63 (11)	C4—C5—H5	119.7
N1—Cu1—O2	82.55 (12)	C5—C6—C1	120.7 (4)
N2—Cu1—O2	91.59 (12)	C5—C6—H6	119.6
O1—Cu1—S1^i^	112.18 (12)	C1—C6—H6	119.6
O2—Cu1—S1^i^	87.96 (12)	N1—C7—C1	125.7 (3)
N1—Cu1—S1^i^	87.62 (12)	N1—C7—H7	117.1
N2—Cu1—S1^i^	87.45 (12)	C1—C7—H7	117.1
C7—N1—C8	120.9 (3)	N1—C8—C9	107.2 (3)
C7—N1—Cu1	125.7 (3)	N1—C8—H8A	110.3
C8—N1—Cu1	113.2 (2)	C9—C8—H8A	110.3
C11—N2—Cu1	171.1 (3)	N1—C8—H8B	110.3
C2—O1—Cu1	125.6 (2)	C9—C8—H8B	110.3
C9—O2—Cu1	110.9 (2)	H8A—C8—H8B	108.5
C9—O2—H2	111 (3)	O2—C9—C8	106.9 (3)
Cu1—O2—H2	107 (3)	O2—C9—H9A	110.3
C3—O3—C10	117.2 (3)	C8—C9—H9A	110.3
C2—C1—C6	120.1 (3)	O2—C9—H9B	110.3
C2—C1—C7	122.8 (3)	C8—C9—H9B	110.3
C6—C1—C7	116.9 (4)	H9A—C9—H9B	108.6
O1—C2—C1	125.3 (3)	O3—C10—H10A	109.5
O1—C2—C3	117.2 (3)	O3—C10—H10B	109.5
C1—C2—C3	117.5 (3)	H10A—C10—H10B	109.5
O3—C3—C4	125.9 (4)	O3—C10—H10C	109.5
O3—C3—C2	113.3 (3)	H10A—C10—H10C	109.5
C4—C3—C2	120.9 (4)	H10B—C10—H10C	109.5
C3—C4—C5	120.2 (4)	N2—C11—S1	179.0 (4)
C3—C4—H4	119.9		

Symmetry codes: (i) *x*, −*y*+1/2, *z*+1/2.

### Hydrogen-bond geometry (Å, °)

**Table:**

*D*—H···*A*	*D*—H	H···*A*	*D*···*A*	*D*—H···*A*
O2—H2···O1^ii^	0.85 (1)	1.96 (2)	2.770 (4)	160 (5)
O2—H2···O3^ii^	0.85 (1)	2.41 (4)	3.020 (4)	129 (4)

Symmetry codes: (ii) −*x*+1, −*y*+1, −*z*.
